# Genetic deficiency of Syk protects mice from autoantibody-induced arthritis

**DOI:** 10.1002/art.27438

**Published:** 2010-07

**Authors:** Zoltán Jakus, Edina Simon, Bálint Balázs, Attila Mócsai

**Affiliations:** Semmelweis University School of MedicineBudapest, Hungary

## Abstract

**Objective:**

The Syk tyrosine kinase plays an important role in diverse functions in hematopoietic lineage cells. Although previous in vitro and pharmacologic analyses suggested Syk to be a possible player in the development of autoimmune arthritis, no in vivo genetic studies addressing that issue have yet been reported. The aim of the present study was to test whether genetic deficiency of Syk affects autoantibody-induced experimental arthritis in the K/BxN serum–transfer model.

**Methods:**

Syk^−/−^ bone marrow chimeras carrying a Syk-deficient hematopoietic system were generated by transplanting Syk^−/−^ fetal liver cells into lethally irradiated wild-type recipients. After complete repopulation of the hematopoietic compartment, autoantibody-mediated arthritis was induced by injection of arthritogenic K/BxN serum. Arthritis development was monitored by macroscopic and microscopic observation of the ankle joints, micro–computed tomography of bone morphology, as well as a joint function assay.

**Results:**

Genetic deficiency of Syk in the hematopoietic compartment completely blocked the development of all macroscopic and microscopic signs of arthritis. The Syk^−/−^ mutation also prevented the appearance of periarticular bone erosions. Finally, Syk^−/−^ bone marrow chimeras were completely protected from arthritis-induced loss of articular function.

**Conclusion:**

Our results indicate that Syk is critically involved in the development of all clinically relevant aspects of autoantibody-mediated K/BxN serum–transfer arthritis in experimental mice. These results provide the first in vivo genetic evidence of the role of Syk in the development of autoimmune arthritis.

Rheumatoid arthritis (RA) is a severe, chronic autoimmune inflammatory disease affecting nearly 1% of the human population (1). The requirement for better and more cost-effective treatment strategies points to the need for a deeper understanding of the disease pathogenesis at the molecular level.

Autoimmune arthritis develops in 2 consecutive phases in experimental animals, and based on indirect (e.g., genetic) evidence, a similar scenario is expected to apply to RA in humans. During the first (“initiation”) phase, genetic and environmental factors lead to the emergence of autoreactive T lymphocytes. During the second (“effector”) phase, those autoreactive T cells lead to synovial inflammation, proliferation, and bone resorption through hematopoietic lineage cells and synovial fibroblasts. The coupling between these 2 phases likely involves autoantibody formation, as well as activation of cytokine networks (e.g., tumor necrosis factor [TNF], interleukin-17 [IL-17]) (2). The reemerging pathogenetic role of autoantibodies is supported by the supposedly proarthritic nature of anti–cyclic citrullinated peptide antibodies (3,4), the beneficial effect of B cell depletion in human RA (5,6), and the capability of autoantibodies to induce autoimmune arthritis in experimental animals (7–9).

The K/BxN arthritis model is a widely used transgenic mouse model of human RA. The peculiarity of this model is that the disease can be transferred to nonarthritic recipients by either the serum or the purified immunoglobulin fraction derived from arthritic K/BxN mice (called K/BxN serum–transfer arthritis), allowing the separate analysis of the autoantibody-mediated effector phase of the disease. Indeed, K/BxN serum–transfer arthritis proceeds normally in RAG-1^−/−^ animals that lack both T and B lymphocytes (7). Further analyses have revealed that K/BxN serum–transfer arthritis is mediated by different myeloid lineage cells (10–12) and the alternative pathway of complement activation (13). This model also requires immune complex recognition by Fcγ receptors (13,14), as well as members of the β_2_ integrin family (15).

Syk is a nonreceptor tyrosine kinase involved in diverse biologic functions, including immunoreceptor (lymphocyte antigen receptor and Fc receptor) signaling (16–20), certain integrin signal transduction processes (21,22), osteoclast development and function (23,24), vascular development (25), or innate immune recognition (26,27). While the functional role of Syk has been extensively tested in a number of various in vitro cellular assays, little is known about its role in live animals and in vivo models of human diseases. This is likely due to the perinatal lethality caused by Syk deficiency (16,17) precluding the analysis of adult Syk^−/−^ animals.

Recently, R406, a small-molecule inhibitor, was identified and shown to be a potent inhibitor of Syk and of a number of supposedly Syk-dependent cellular responses of various lymphoid and myeloid lineage cells (28). Importantly, R406 attenuated autoantibody-induced arthritis in mice (28), whereas its orally bioavailable prodrug form R788, or fostamatinib, inhibited collagen-induced arthritis in rats (29). Initial clinical analysis of fostamatinib in RA also revealed significant clinical benefit in patients receiving methotrexate therapy (30), as well as in those whose RA previously failed to respond to methotrexate therapy alone (http://www.rigel.com/pdf/R788TASKI2-3RAResults.pdf). Those results suggest that fostamatinib may be exploited as an oral antirheumatic agent in the future.

While the in vivo effect of R406 (and its fostamatinib prodrug) on arthritis development is well documented, its selectivity for Syk is somewhat questionable. The original conclusion that Syk is the primary target of R406 was based on rather indirect evidence, and the primary results of an in vitro kinase selectivity profiling have not yet been published (28). While R406 exerted half-maximal inhibition of Syk at 30 n*M* (28), it inhibited the Flt-3 and Ret tyrosine kinases at <10 n*M* (31,32). R406 also inhibited c-Kit, Lck, JAK-1/3, and the adenosine A_3_ receptor in the mid-nanomolar concentration range (28). Most of those potential alternative R406 targets are involved in inflammation and autoimmunity (33–37), providing a possible alternative explanation for the beneficial effect of R406 on arthritis. Therefore, a more specific (e.g., genetic) approach would be required to unequivocally conclude that Syk is involved in arthritis development.

Even if one assumes that R406 and fostamatinib only act on Syk, a number of additional questions related to the role of Syk in arthritis remain. First, those inhibitors only partially decreased disease development in animal models of autoimmune arthritis (28,29). It is, at present, unclear whether the remaining disease activity was due to an incomplete inhibition of Syk or was due to a parallel, Syk-independent mechanism. Second, it is mostly unclear whether Syk participates in signaling within hematopoietic or nonhematopoietic cells during arthritis development. Indeed, while Syk is mostly expressed in the hematopoietic compartment, recent studies have suggested that it may also play a functional role in synovial fibroblasts (38,39). Those issues could most easily be addressed by analyzing the genetic deficiency of Syk, preferably in an at least partially lineage-restricted manner.

The above issues prompted us to test the role of Syk in the pathogenesis of autoimmune arthritis by using a genetic approach, that is, by testing the development of the autoantibody-induced K/BxN serum–transfer arthritis in chimeric mice with a Syk-deficient hematopoietic compartment. Our results indicate that Syk present in the hematopoietic compartment is indispensable for the autoantibody-mediated component of autoimmune arthritis.

## MATERIALS AND METHODS

### Animals

Heterozygous mice carrying a deleted Syk allele (Syk^tm1Tyb^, which is referred to as Syk^−^) (16) were obtained from Victor Tybulewicz (National Institute for Medical Research, London, UK). The mutation was maintained in heterozygous (Syk^+/−^) form on the C57BL/6 genetic background (i.e., carrying the CD45.2 allele). Mice carrying the KRN T cell receptor (TCR) transgene (40) were obtained from Diane Mathis and Christophe Benoist (Harvard Medical School, Boston, MA) and were maintained in heterozygous form by mating with C57BL/6 mice. KRN transgene–positive mice were identified by flow cytometric analysis of the expression of the V_β_6 TCR (40,41). NOD mice as well as a congenic strain carrying the CD45.1 allele on the C57BL/6 genetic background (B6.SJL-*Ptprc*^a^) were purchased from The Jackson Laboratory. Mice (4–6 per group) were maintained in individually sterile ventilated cages (Tecniplast) in a conventional facility. All animal experiments were approved by the Semmelweis University Animal Experimentation Review Board.

### Bone marrow transplantation

**Figure 1:**
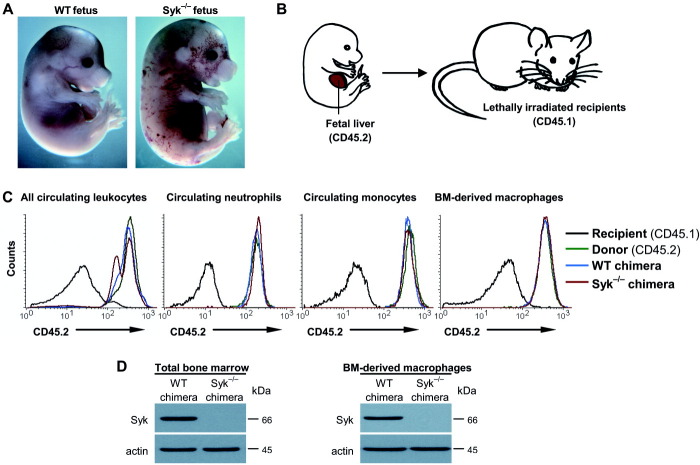
Generation and analysis of Syk^−/−^ bone marrow chimeric mice. **A**, Macroscopic appearance of wild-type (WT) and Syk^−/−^ mouse fetuses at ∼17.5 days postcoitum (embryogenesis day 17.5). **B**, General scheme of fetal liver transplantation procedure. **C**, Flow cytometric analysis of donor marker (CD45.2) expression in all circulating leukocytes (CD45+ gate), circulating neutrophils (Gr-1+ gate), circulating monocytes (CD11b+Gr-1– gate), and bone marrow (BM)–derived macrophages (F4/80+ gate) from intact (nonchimeric) mice of the CD45.1-expressing recipient strain or the CD45.2-expressing donor (C57BL/6) strain, as well as from wild-type or Syk^−/−^ bone marrow chimeras. **D**, Immunoblot analysis of Syk and β-actin protein levels in total bone marrow cells or bone marrow–derived macrophages. Results are representative of 3 or more independent experiments, each of which showed similar results. Actin was used as a loading control.

Bone marrow chimeras with the Syk^−/−^ hematopoietic system were generated by fetal liver transplantation using fetuses from days 15.5–18.5 of embryogenesis (E15.5–18.5), which were obtained from timed matings of Syk^+/−^ carriers. The 8–16-week-old recipient mice carrying the CD45.1 allele on the C57BL/6 genetic background were lethally irradiated as described (41) and then injected intravenously with unfractionated fetal liver cell suspensions. On average, fetal liver cells from a single donor were injected into 5–8 recipients. Syk^−/−^ fetuses were identified according to their characteristic petechiated appearance (16,17) (Figure 1A), and their genotype was occasionally confirmed by allele-specific polymerase chain reaction analysis (21). An equal number of control chimeras were also generated using macroscopically normal (Syk^+/+^ or Syk^+/−^) sibling fetuses. Since our initial experiments did not reveal any differences between K/BxN serum–transfer arthritis in the Syk^+/+^ and the Syk^+/−^ mice (see below), no further distinction between those 2 genotypes was made, and we refer to both the Syk^+/+^ and the Syk^+/−^ bone marrow chimeras as wild-type chimeras.

### Assessment of chimerism

Peripheral blood samples were taken 4–6 weeks after transplantation and stained with phycoerythrin (PE)–labeled antibodies against CD45 (clone 30-F11, which recognizes all forms of CD45) or against Gr-1 (clone RB6-8C5), or were stained with biotin-conjugated antibodies against CD11b (clone M1/70), the latter followed by streptavidin–PerCP staining. Primary bone marrow–derived macrophages were obtained as described previously (42) and were stained with PE-labeled antibodies against the F4/80 antigen (clone CI:A3-1). All samples were counterstained with fluorescein isothiocyanate–labeled antibodies against the donor-specific CD45.2 epitope (clone 104). Except for the anti-F4/80 antibody, which was purchased from AbD Serotec, all antibodies were obtained from BD Biosciences. Flow cytometry was performed using a FACSCalibur instrument, and data were analyzed using CellQuest software (both from BD Biosciences). The various leukocyte subsets were identified based on their forward and side scatter characteristics and the expression of the indicated lineage-specific markers, followed by the assessment of the expression of the donor-specific CD45.2 epitope.

For the analysis of Syk protein levels, total bone marrow cells or bone marrow–derived macrophages were immunoblotted using anti-Syk (N-19; Santa Cruz Biotechnology) or anti–β-actin (clone AC-74; Sigma) antibodies as described elsewhere (42).

### K/BxN serum–transfer arthritis

Mice carrying the KRN TCR transgene (40) on the C57BL/6 background were mated with NOD mice to obtain transgene-positive K/BxN mice as well as their transgene-negative (BxN) littermates. The presence of the KRN transgene was determined by flow cytometric analysis of the expression of the V_β_6 TCR in circulating CD4 T cells as well as by looking for visible signs of arthritis in the K/BxN mice, as described previously (40,41). Blood was taken by retroorbital bleeding, and sera from KRN transgene–positive and transgene–negative mice were pooled separately.

Arthritis was induced by a single intraperitoneal injection of 400 μl of arthritogenic (K/BxN) or control (BxN) serum on day 0 of the experiment. Each experimental group contained chimeras generated using 1–2 donor fetal livers. Chimeras generated using a total of 11 Syk^−/−^ and an equal number of wild-type control fetal livers were used during the course of this study.

### Assessment of arthritis

Arthritis severity in each mouse was assessed daily for 2 weeks following serum injection. Visible signs of arthritis were scored on a scale of 0–10 by 2 investigators who were blinded as to the origin and treatment of the mice (41). Ankle thickness was measured with spring-loaded calipers (Kroeplin). Photographs were taken on day 8 of the disease.

To assess articular function, mice were placed on a custom-made wire grid that was then flipped upside down, and the length of time the mice held on to the wire grid during a 20-second assessment period was recorded (41). This test was performed several times daily during the plateau phase of the disease (days 8–12). The data obtained were combined into “holding-on curves” similar to Kaplan-Meier survival curves.

Mice used in histologic analyses were killed on day 4, their ankle joints were harvested, fixed in 10% formalin, decalcified in OsteoMoll decalcifying solution (Merck Chemicals), embedded in paraffin, sectioned, and then stained with hematoxylin and eosin. Photomicrographs were taken with a Leica DMI 6000B inverted microscope.

### Micro–computed tomography (micro-CT) analysis

Mice used in the micro-CT analyses were killed on day 8, the skin was removed, and the ankles were placed in phosphate buffered saline containing 0.1% sodium azide. The ankles were scanned using a SkyScan 1172 micro-CT apparatus operating at a resolution of 9 μm voxel size. Three-dimensional reconstruction was performed using NRecon software, and the images were further processed with CTanalyser software.

Once micro-CT analyses of the entire ankles were completed, the tissues were digested overnight at 55°C in 0.2 mg/ml of proteinase K (Roche) in the presence of 1% sodium dodecyl sulfate and 2 m*M* CaCl_2_. The first distal tarsal bones were then identified and rescanned at a resolution of 5 μm voxel size.

### Presentation of the data and statistical analysis

Results are expressed as the mean ± SD of the indicated number of individual data points. For the sake of clarity (i.e., to avoid overlapping symbols and error bars), every second data point was omitted from the presentation. For statistical analysis of arthritis severity, average values obtained from the control serum–treated animals of the same genotype in the same experiment were subtracted from those of the arthritic serum–treated animals. To avoid calculating with multiple values from a single mouse, values for the left and right limbs in studies of macroscopic arthritis were averaged, whereas data from a single mouse in the joint function assays were combined into individual holding-on curves before further analysis of statistical significance. The data thus obtained were then compared across the different genotypes by use of the nonparametric Mann-Whitney U test using Statistica software (StatSoft). Arthritis severity was analyzed on day 9. Joint function was assessed at the 20-second time point. Exact *P* values less than 0.05 were considered to be statistically significant.

## RESULTS

### Generation of Syk^−/−^ bone marrow chimeras and assessment of chimerism

Complete genetic deficiency of Syk leads to a petechiated in utero appearance (Figure 1A) and perinatal lethality (16,17), which are likely due to a lymphatic vascular developmental defect (25). This problem was overcome by the generation of bone marrow chimeras with a Syk^−/−^ hematopoietic system in an otherwise normal nonhematopoietic environment (21). To this end, Syk^−/−^ fetal liver cells were injected intravenously into lethally irradiated recipient mice (Figure 1B). An equal number of Syk-sufficient (Syk^+/+^ or Syk^+/−^) control chimeras were also generated; these are referred to herein as wild-type chimeras. Recipients were chosen to carry the CD45.1 allele, which can be distinguished from the donor-derived CD45.2 allele by patterns on flow cytometry. The generated Syk^−/−^ bone marrow chimeras did not show any overt phenotype during the course of this study (usually, 4–10 weeks after transplantation).

To confirm the complete replacement of the hematopoietic compartment, we tested the presence of donor-specific markers in bone marrow chimeras. As shown in Figure 1C, circulating leukocytes (i.e., cells expressing the pan-leukocyte marker CD45) of both wild-type and Syk^−/−^ chimeras were nearly exclusively of donor origin (CD45.2+). Since K/BxN serum–transfer arthritis requires various myeloid cell types (10,11), we also analyzed various myeloid lineages. As shown in Figure 1C, circulating neutrophils (defined as Gr-1+ cells) and monocytes (CD11b+Gr-1– cells) from peripheral blood samples, as well as bone marrow–derived macrophages (F4/80+ cells) of both wild-type and Syk^−/−^ chimeras were all exclusively of donor origin (CD45.2+). Additional experiments in which Syk protein expression was analyzed by immunoblotting revealed that Syk was absent from lysates of total bone marrow cells or bone marrow–derived macrophages from Syk^−/−^ chimeras, but not from parallel generated wild-type control chimeras (Figure 1D).

Taken together, the above transplantation approach allowed us to generate bone marrow chimeras with a Syk^−/−^ hematopoietic compartment.

### Protection of Syk^−/−^ chimeras from clinical signs of autoantibody-induced arthritis

**Figure 2:**
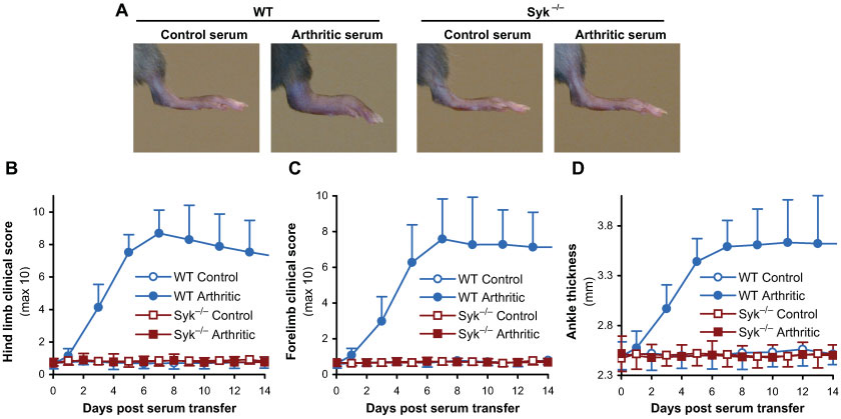
Assessment of the macroscopic signs of arthritis in Syk^−/−^ bone marrow chimeric mice. **A**, Photographs of the hind limb of wild-type (WT) or Syk^−/−^ chimeras 8 days after a single injection of 400 μl of arthritogenic (K/BxN) or control (BxN) serum. **B–D**, Quantification of arthritis severity by hind limb (**B**) or fore limb (**C**) clinical scoring or by measurement of ankle thickness (**D**). Experiments were performed on all 4 limbs of 6 control serum– and 11 arthritogenic serum–treated mice per genotype in 4 independent experiments. Shown are representative photographs (**A**) or the mean and SD values (**B–D**) of 2 limbs per mouse from all mice tested.

To test the contribution of Syk to K/BxN serum–transfer arthritis, wild-type and Syk^−/−^ chimeras were injected with arthritogenic (K/BxN) serum or control serum. While arthritogenic serum triggered severe inflammation of the hind paws of wild-type bone marrow chimeras, no signs of the disease were seen in similarly treated Syk^−/−^ chimeras (Figure 2A). Quantification of hind paw arthritis by clinical scoring also revealed severe disease in wild-type bone marrow chimeras injected with arthritogenic K/BxN serum, whereas no disease was seen in similarly treated Syk^−/−^ chimeras (Figure 2B). Compared with the clinical scores in control serum–injected mice, injection of arthritogenic serum caused a marked increase (mean ± SD 7.5 ± 2.2 points) in the clinical score in wild-type chimeras on day 9, whereas the same treatment caused a negligible decrease (0.02 ± 0.2 points) in Syk^−/−^ chimeras. The difference between the 2 genotypes was highly statistically significant (*P* = 1.5 × 10^−6^; n = 11). A complete lack of arthritis development was also observed in the fore limbs of Syk^−/−^ chimeras (Figure 2C).

Ankle thickness measurements also indicated a robust thickening of the ankles of arthritogenic serum–injected wild-type bone marrow chimeras, whereas no increase was seen in similarly treated Syk^−/−^chimeras (Figure 2D). Quantitative analyses indicated that injection of arthritogenic serum increased the ankle thickness in wild-type chimeras by a mean ± SD of 1.1 ± 0.3 mm over that in control serum–treated animals on day 9, whereas the same treatment led to a negligible increase of 0.01 ± 0.1 mm in Syk^−/−^ chimeras. The difference between the 2 genotypes was again highly significant (*P* = 1.5 × 10^−6^; n = 11).

Taken together, the data indicate that hematopoietic expression of Syk is indispensable for the development of clinical signs of arthritis in the K/BxN serum–transfer model.

### Normal course of K/BxN serum–transfer arthritis in heterozygous Syk^+/−^ animals

**Figure 3:**
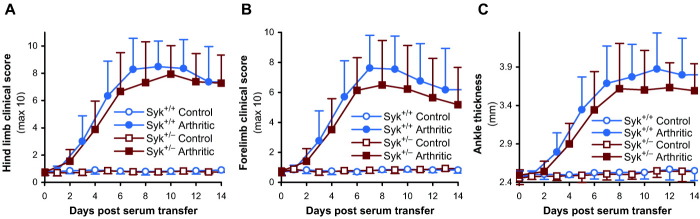
Normal arthritis development in heterozygous Syk^+/−^ mice. Arthritis severity was quantified by hind limb (**A**) or fore limb (**B**) clinical scoring or by measurement of ankle thickness (**C**) in intact (nonchimeric) homozygous Syk^+/+^ or heterozygous Syk^+/−^ mice after a single injection of 400 μl of arthritogenic (K/BxN) or control (BxN) serum. Experiments were performed on all 4 limbs of 5 control serum– and 10 arthritogenic serum–treated mice per genotype in 3 independent experiments. Values are the mean and SD of 2 limbs per mouse from all tested mice combined.

The preceding experiments tested the effect of the complete deletion of Syk in the hematopoietic compartment. We next investigated whether the partial reduction of Syk expression affects the development of autoantibody-induced arthritis. To this end, we compared the severity of K/BxN serum–transfer arthritis in intact (nonchimeric) Syk^+/+^ and Syk^+/−^ littermates. Clinical scores in the hind limbs (Figure 3A) did not reveal any significant differences in disease severity between the 2 genotypes (clinical score on day 9 increased by a mean ± SD of 7.7 ± 1.4 and 6.8 ± 1.9 in Syk^+/+^ and Syk^+/−^ mice, respectively [*P* = 0.24]; n = 10), nor were any considerable differences in clinical scores in the fore limbs seen between the 2 genotypes (Figure 3B). Similarly, the Syk^+/−^ mutation did not significantly affect ankle thickening (ankle thickness on day 9 increased by 1.3 ± 0.3 and 1.0 ± 0.3 mm in Syk^+/+^ and Syk^+/−^ mice, respectively [*P* = 0.11]; n = 10) (Figure 3C). Those results indicate that approximately half of the Syk kinase activity is sufficient to support mostly normal development of autoantibody-induced arthritis.

### Histologic analysis of arthritis in Syk^−/−^ bone marrow chimeras

**Figure 4:**
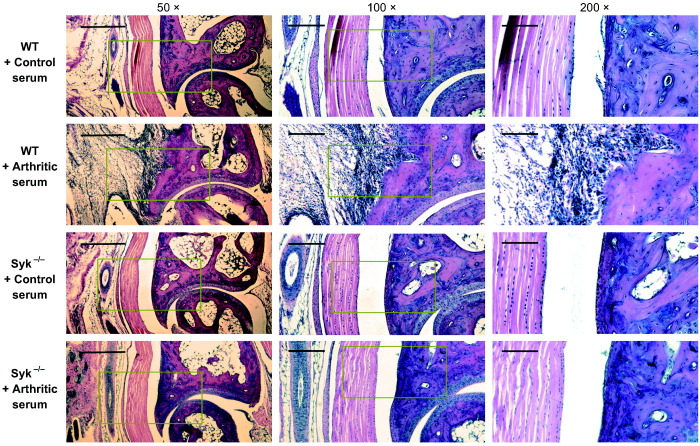
Histologic analysis of the ankle joints of Syk^−/−^ bone marrow chimeric mice. Photomicrographs of hematoxylin and eosin–stained sagittal sections of the ankle joints of wild-type (WT) or Syk^−/−^ chimeras 4 days after a single injection of 400 μl of arthritogenic (K/BxN) or control (BxN) serum are shown. In each case, sections obtained at 50× magnification show the joint formed between the tibia (above) and the astragalus (below) bones (the latter corresponding to the human talus), with the posterior surface pointing to the left. Boxed areas in images shown at 50× magnification are shown at 100× magnification in images in the center column, and boxed areas in images shown at 100× magnification are shown at 200× magnification in images in the right column. Bars = 500 μm, 200 μm, and 100 μm for 50×, 100×, and 200× magnification, respectively. Images are representative of a large number of sections obtained from 3 independent experiments, each of which showed similar results.

Next, we performed histologic analysis of arthritis development in the chimeras. Hematoxylin and eosin–stained sections of the joints obtained 4 days after serum injection showed that between the tibia and astragalus bones (the latter corresponding to the talus in humans), arthritogenic serum induced a robust leukocytic infiltration of the periarticular tissues in wild-type chimeras (Figure 4). This infiltrate even eroded the marginal areas of the articular surface and invaded the juxtaarticular free (non–joint-forming) bone surface of the tibia. Importantly, no leukocytic infiltration or erosion was seen in Syk^−/−^ bone marrow chimeras that had been injected with arthritogenic serum (Figure 4). Hence, Syk in the hematopoietic compartment is required for the development of microscopic signs of arthritis, such as leukocyte accumulation or periarticular erosion of the bone and cartilage surface.

### Lack of periarticular bone erosions in Syk^−/−^ bone marrow chimeras

**Figure 5:**
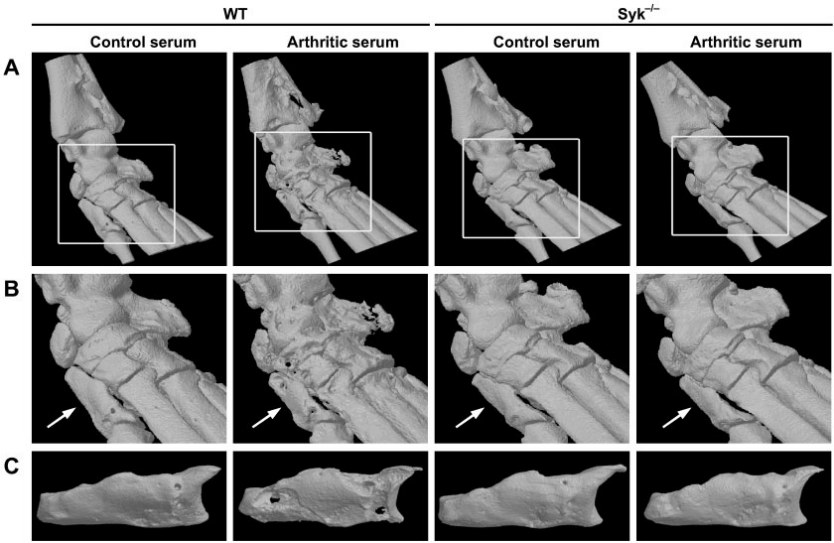
Micro–computed tomography (micro-CT) of the ankle joints of Syk^−/−^ bone marrow chimeric mice. **A** and **B**, Three-dimensional reconstruction of the ankle bones of wild-type (WT) and Syk^−/−^ bone marrow chimeras obtained by micro-CT at 9 μm voxel size on day 8 after a single injection of 400 μl of arthritogenic (K/BxN) or control (BxN) serum. Boxed areas in **A** are shown at higher digital magnification in **B**. **Arrow** in **B** indicates the first distal tarsal bone. **C**, Three-dimensional images of the first distal tarsal bone obtained by micro-CT at 5 μm voxel size after complete digestion of the ankle joints. Images are representative of 3 ankles per group obtained from 3 independent experiments, each of which showed similar results.

Arthritis-induced periarticular bone erosions lead to irreversible loss of the joint architecture and indicate a poor prognosis of the disease. Arthritis-induced bone erosions result from the activity of osteoclasts (43–46), which have previously been shown to rely on Syk for their development and function (23,24). To obtain a detailed picture of the erosion of mineralized bone tissue, we performed micro-CT analysis of the ankle area of our bone marrow chimeras. As shown in Figures 5A and B, injection of arthritogenic serum induced marked erosions of the periarticular bone surfaces in wild-type chimeras, even punching holes across several tarsal bones. In contrast, the periarticular bone surfaces of similarly treated Syk^−/−^ chimeras were smooth and unaffected, similar to those of control serum–treated animals (Figures 5A and B).

A more-detailed analysis of the first distal tarsal bone (equivalent of the first cuneiform bone in humans) (white arrows in Figure 5B) after proteolytic digestion of the ankle showed extensive surface erosions and several holes across the entire bone in arthritogenic serum–injected wild-type chimeras (Figure 5C). In contrast, the architecture and bone surface of the first distal tarsal bone of similarly treated Syk^−/−^ chimeras did not show any signs of bone erosion.

Taken together, these findings indicate that Syk in the hematopoietic compartment is required for the development of periarticular bone erosions during autoantibody-induced arthritis.

### Protection of Syk^−/−^ bone marrow chimeras from arthritis-induced loss of articular function

**Figure 6:**
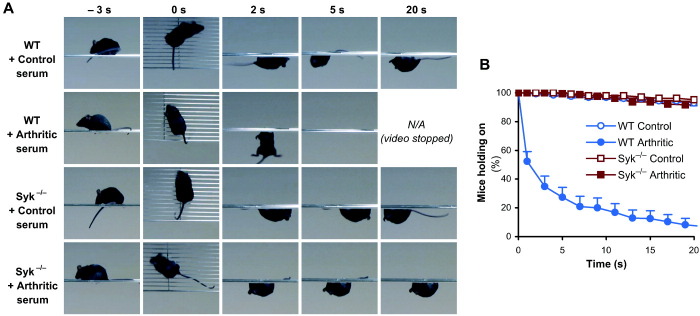
Protection of Syk^−/−^ bone marrow chimeric mice from arthritis-induced loss of articular function. Wild-type (WT) or Syk^−/−^ bone marrow chimeras were injected with a single dose of 400 μl of arthritogenic (K/BxN) or control (BxN) serum. On days 8–12 after serum injection, the mice were placed on a custom-made wire grid, which was then flipped over, and the length of time the mice held on to the wire grid during a 20-second assessment period was recorded. **A**, Snapshots from the video captures of mice of the indicated genotype and treatment groups 10 days after serum injection. **B**, Percentages of mice that were able to hold on to the grid at the indicated time points after the grid was flipped over. The experiment was repeated 15–20 times per mouse (n = 6 control serum–treated and n = 11 arthritic serum–treated chimeras per genotype in 4 independent experiments). Shown are representative snapshots (**A**) or the mean and SD values (**B**) for each group of mice tested.

Besides macroscopic and microscopic signs of inflammation, arthritis also leads to severe impairment of articular function. This was assessed by testing the ability of our chimeras to hold on to the bottom of a horizontal wire grid (41). As shown in the video snapshots in Figure 6A, control serum–treated chimeras held on to the grid for the entire 20-second period, whereas wild-type chimeras injected with arthritogenic serum fell off the grid within a few seconds, indicating an arthritis-induced loss of articular function. Importantly, Syk^−/−^ bone marrow chimeras injected with arthritogenic serum were able to hold on to the wire grid for the entire period of assessment (Figure 6A).

The above experiment was repeated several times on each mouse 8–12 days after serum injection, and the percentages of mice that were able to hold on to the grid for a given period were calculated analogous to the calculation of Kaplan-Meier survival curves. While more than 90% of control serum–treated chimeras held on to the wire grid for the entire 20-second assessment period, only ∼10% of the wild-type chimeras injected with arthritogenic serum were able to do so (Figure 6B). Importantly, arthritogenic serum–treated Syk^−/−^ chimeras held on to the wire grid as well as the control serum–injected animals had (Figure 6B). Statistical analysis revealed that, compared with control serum–treated animals, treatment with arthritogenic serum caused an additional 85 ± 16% and 3.4 ± 7.6% (mean ± SD) of the wild-type and Syk^−/−^ chimeras, respectively, to fall off the grid during the 20-second assessment period (*P* = 1.5 × 10^−6^; n = 11).

Taken together, the findings show that genetic deficiency of Syk in the hematopoietic compartment protects mice from arthritis-induced loss of articular function.

## DISCUSSION

Development of better, safer, and cheaper therapeutic strategies for RA would require better understanding of the pathologic mechanism of the disease at the molecular level. While previous pharmacologic studies have suggested a role for the Syk tyrosine kinase in the development of autoimmune arthritis, all of those studies were based on a single pharmacologic agent with limited specificity for Syk. Using mice that are genetically deficient in Syk, our experiments showed that Syk within the hematopoietic compartment is required for autoantibody-induced arthritis to occur in the K/BxN serum–transfer model. These studies provide the first genetic evidence of the role of Syk in the pathogenesis of autoimmune arthritis.

During the last decade, tyrosine kinase inhibitors have been demonstrated to have major therapeutic potential in diverse diseases, particularly tyrosine kinase–dependent tumors. While most of those drugs were originally considered to be specific for a particular kinase, later studies revealed that they also affect other tyrosine phosphorylation pathways. Imatinib, for example, was originally developed to inhibit the breakpoint cluster region (BCR)-Abl fusion protein, but it was later shown to also inhibit c-Kit and the platelet-derived growth factor receptor. Similarly, dasatinib inhibits BCR-Abl fusion protein and Src family kinases, but it also affects other kinases, such as c-Kit or the ephrins. Indeed, it has proved rather difficult to specifically target a given kinase, especially because most of these drugs act as competitive inhibitors at the highly conserved ATP-binding pocket. However, despite their limited specificity, these drugs clearly provide major clinical benefit in various groups of patients, indicating that specificity for a single kinase is not necessarily a prerequisite for the clinical usefulness of a tyrosine kinase inhibitor. Indeed, a particular spectrum of different targets (“multiple targeting”) may actually be more beneficial in a given clinical setting.

Fostamatinib (R788), an orally bioavailable prodrug of the R406 tyrosine kinase inhibitor, has been developed for the treatment of allergic and inflammatory diseases, and it has recently been found to provide significant therapeutic benefit in certain RA patients as well (see ref.^30^ and http://www.rigel.com/pdf/R788TASKI2-3RAResults.pdf). R406 has been shown to be a potent inhibitor of Syk, acting as a competitive inhibitor at the ATP-binding site (28). However, additional studies revealed that it also affects other targets, including the Flt-3, Ret, c-Kit, Lck, and JAK-1/3 tyrosine kinases, the adenosine A_3_ receptor, as well as several additional kinases and nonkinase targets, often at concentrations comparable to, or even lower than, those inhibiting Syk (28,31,32). The role of most of those potential R406 targets in inflammation and autoimmunity (33–37) could even provide an alternative explanation for the effect of R406 in arthritis development. Taken together, R406/fostamatinib should not be considered a specific inhibitor of Syk tyrosine kinase, and the beneficial effect of R406 or fostamatinib on autoimmune arthritides should not be considered formal proof of the role of Syk in the pathogenesis of those diseases.

By using an approach that is independent of, and conceptually different from, the above pharmacologic approach, our genetic studies presented herein provide independent evidence of the role of Syk in the autoantibody-mediated component of the effector phase of autoimmune arthritis. Together with the findings of the above pharmacologic studies, we can now confidently conclude that Syk is indeed an important component of at least certain aspects of the pathogenesis of autoimmune arthritis.

Besides providing the first genetic evidence for the role of Syk in arthritis, our results also allow a more detailed understanding of how Syk participates in arthritis development. Previous studies showing a partial reduction of arthritis severity by treatment with R406 or fostamatinib in animal models of arthritis, including the K/BxN serum–transfer arthritis model (28,29), raised the possibility that the lack of Syk activity can be partially compensated for by another, Syk-independent mechanism. Our results showing complete protection of Syk^−/−^ bone marrow chimeras from arthritis development in the K/BxN serum–transfer model indicate that, at least in the case of autoantibody-induced arthritis, no Syk-independent component exists. Previous pharmacologic studies could not address whether the presence of Syk is required in hematopoietic or nonhematopoietic cells to support arthritis development. Indeed, while the primarily hematopoietic expression of Syk supported the former scenario, recent studies have also suggested a role of Syk in synovial fibroblasts of RA patients (38,39). Our results obtained in studies of chimeric mice lacking Syk only in the hematopoietic compartment indicate that Syk within the hematopoietic compartment is indispensable for the autoantibody-mediated component of autoimmune arthritis (though it does not exclude the possibility of a parallel role of Syk in synovial fibroblasts).

RA is a complex disease with various consecutive phases and several parallel signaling mechanisms. While our studies identified Syk as a critical player in the autoantibody-induced component of autoimmune arthritis, no conclusion about the role of Syk in other components of the disease, such as autoantibody formation or cytokine (TNF, IL-17, etc.)–mediated activation of effector cells, should be drawn (2). Instead, these questions need to be addressed by other appropriate approaches, such as analysis of Syk^−/−^ bone marrow chimeras, in additional experimental models of arthritis.

It has been proposed that K/BxN serum–transfer arthritis is mediated by a number of nonlymphoid lineages of hematopoietic origin. Given the critical role of neutrophils in K/BxN serum–transfer arthritis (10) and the requirement for Syk in integrin (21) and Fc receptor (18) signaling in neutrophils, we expect that Syk in neutrophils makes a major contribution to the observations described herein. We are less certain about the functional role of Syk in other hematopoietic lineages. While macrophages also rely on Syk for signaling by integrins (47) and Fcγ receptors (18,48), their contribution to arthritis development in the K/BxN serum–transfer model is a subject of some controversy (compare ref.^11^ with ref.^49^). There are controversies related to the role of mast cells in experimental arthritis as well (compare refs.^12^ and^50^ with ref.^51^), and our bone marrow transplantation approach is unlikely to replace all tissue mast cells, given the inherent radioresistance of that cell lineage (52). While we and other investigators have shown that Syk is required for osteoclast development and function (23,24), we cannot exclude the possibility that the lack of bone erosions in Syk^−/−^ bone marrow chimeras is secondary to the defective process of inflammation, rather than being due to a cell-autonomous defect of Syk^−/−^ osteoclasts in the resorption of bone in an inflammatory environment. Finally, as mentioned above, our experimental approach cannot provide any information on the role of Syk within cells of nonhematopoietic origin. Obviously, further lineage-specific studies will be needed in order to identify the lineage(s) requiring Syk for signaling during the development of autoimmune arthritis.

Although initial studies of Syk suggested that it is primarily involved in signaling by classic immunoreceptors (20), later studies also indicated its role in signaling by integrins (21,53) and C-type lectins (27,54), as well as in the activation of the NLRP3 inflammasome (26). Of those pathways, Fc receptors (13,14) and β_2_ integrins (15) are well known for their role in the K/BxN serum–transfer arthritis, and various forms of arthritis have also been associated with C-type lectins (55) and the NLRP3 inflammasome (56,57). Hence, there are a number of possible intracellular signaling pathways that may rely on Syk to promote the development of autoimmune arthritis.

An interesting observation of our study was the complete absence of joint-infiltrating leukocytes in Syk^−/−^ bone marrow chimeras. While the role of Syk in integrin signaling would suggest a cell-autonomous defect of β_2_ integrin–mediated leukocyte migration (compare ref.^21^ with ref.^12^), results of previous observations of the normal accumulation of Syk^−/−^ neutrophils in vivo (21,58) are evidence against that possibility. A more likely scenario is that the genetic deficiency of Syk prevents the development of a proper inflammatory environment, leading to the failure of leukocyte migration because of the lack of a migration-promoting cytokine and chemokine milieu, rather than because of a cell-autonomous migration defect.

Taken together, our results provide the first in vivo genetic evidence of the role of Syk in the development of autoimmune arthritis in mice and provide important insight into how Syk participates in the disease. However, further detailed experiments will be required to elucidate other aspects of the role of Syk in arthritis development that could not be addressed in this project.

## AUTHOR CONTRIBUTIONS

All authors were involved in drafting the article or revising it critically for important intellectual content, and all authors approved the final version to be published. Dr. Mócsai had full access to all of the data in the study and takes responsibility for the integrity of the data and the accuracy of the data analysis.

**Study conception and design.** Jakus, Mócsai.

**Acquisition of data.** Jakus, Simon, Balázs.

**Analysis and interpretation of data.** Jakus, Mócsai.
